# Physical Predictors of Cognitive Performance in Healthy Older Adults: A Cross-Sectional Analysis

**DOI:** 10.1371/journal.pone.0070799

**Published:** 2013-07-30

**Authors:** Christiaan G. Blankevoort, Erik J. A. Scherder, Martijn B. Wieling, Tibor Hortobágyi, Wiebo H. Brouwer, Reint H. Geuze, Marieke J. G. van Heuvelen

**Affiliations:** 1 Center for Human Movement Sciences, University of Groningen, University Medical Center Groningen, Groningen, The Netherlands; 2 Department of Neuropsychology, VU University, Amsterdam, The Netherlands; 3 Department of Quantitative Linguistics, University of Tübingen, Tübingen, Germany; 4 Department of Neurology, University of Groningen, University Medical Center Groningen, Groningen, The Netherlands; 5 Department of Clinical and Developmental Neuropsychology, University of Groningen, Groningen, The Netherlands; Federal University of Rio de Janeiro, Brazil

## Abstract

There is ample evidence that physical and cognitive performance are related, but the results of studies investigating this relationship show great variability. Both physical performance and cognitive performance are constructs consisting of several subdomains, but it is presently unknown if the relationship between physical and cognitive performance depends on subdomain of either construct and whether gender and age moderate this relationship. The aim of this study is to identify the strongest physical predictors of cognitive performance, to determine the specificity of these predictors for various cognitive subdomains, and to examine gender and age as potential moderators of the relationship between physical and cognitive performance in a sample of community-dwelling older adults. In total, 98 men and 122 women (average age 74.0±5.6 years) were subjected to a series of performance-based physical fitness and neuropsychological tests. Muscle strength, balance, functional reach, and walking ability (combined score of walking speed and endurance) were considered to predict cognitive performance across several domains (i.e. memory, verbal attention, visual attention, set-shifting, visuo-motor attention, inhibition and intelligence). Results showed that muscle strength was a significant predictor of cognitive performance for men and women. Walking ability and balance were significant predictors of cognitive performance for men, whereas only walking ability was significant for women. We did not find a moderating effect of age, nor did we find support for a differential effect of the physical predictors across different cognitive subdomains. In summary, our results showed a significant relationship between cognitive and physical performance, with a moderating effect of gender.

## Introduction

Demographic data suggest that the number of older adults will increase at an accelerating rate in the coming decades [1]. Age is a risk factor for different aspects of physical performance, such as muscle strength, endurance, and balance [2], [3] and also for impairment in cognition, including episodic memory and executive function (e.g., inhibition, planning, and set-shifting [4]). Although epidemiological studies show a positive relationship between physical performance and cognition [5]–[9], a number of questions remain open [10]. We specifically address three issues that might affect the physical performance-cognition association in older adults: (1) the selection of cognitive domains, (2) gender, and (3) age.

As for test and domain selection, prior studies used a wide variety of methods and tests to quantify the association between physical and cognitive performance. Most prominent is the divergent selection of physical performance domains (e.g., mobility, balance, strength, or endurance) and cognitive performance domains (e.g., memory, global cognitive performance, fluency, attention, or executive functions) across studies to represent physical and cognitive performance [7]–[10]. While the use of many tests and domains is a logical consequence of the desire to assess multiple facets of physical and cognitive performance, this approach also increases the heterogeneity in predicting cognition from motor performance across studies. In addition to the issue of test and domain selection, there are also differences between studies in ethnicity, age, and the number of comorbidities. Due to variations between studies so far, multiple studies need to be taken into account to provide a coherent overview. Unfortunately, the differences between studies make it difficult to determine which physical performance tests are the strongest predictors of (individual measures of) cognitive performance. Indeed, the association between physical and cognitive performance varies widely between studies [10].

Furthermore, it is not well known if gender differences affect the association between measures of physical and cognitive performance. Imaging and neuroanatomical data provide a conceptual basis to expect a gender effect in the association between physical and cognitive performance in community-dwelling older adults [11], [12]. The male brain is larger than the female brain, even after controlling for height, but the decline in volume is also steeper for men than for women [12], [13]. In addition, gender differences in cerebral blood flow after a cognitive task have been observed [14]. More specifically, gender differences are well documented in terms of maximal voluntary leg strength [15], [16] and grip strength [16], [17] in healthy older adults, with women also exhibiting greater reductions in motor coordination [16] than men. With respect to cognition, there is some support for a better overall cognitive performance in aging women versus men [18], especially in memory tasks [19]. Based on these findings it is conceivable that gender differences may influence the association between physical and cognitive performance.

Finally, it is unclear what the effect of age is on the relationship between physical and cognitive performance in healthy older adults. While there is a parallel increase in the variability of physical and cognitive performance with age, the rate of decline differs between the two domains: cognitive impairment accelerates after the age of 60 [4], but the decline in balance and muscle strength accelerates only markedly after the age of 75 [20], [21]. Such a temporal dissociation can confound the associations between physical and cognitive performance in healthy older adults.

The goal of the present study was to re-examine the association between physical and cognitive performance in community-dwelling older men and women. In an effort to better understand the relationship between physical and cognitive performance, we examined this association using a wide array of important physical and cognitive domains which are known to be vulnerable for age-related decline. Concretely, for the physical domain we included measures of gait speed, endurance, grip strength, quadriceps strength, and balance. Each of these measures of physical performance are well-documented in terms of age-related decline [22]–[24] due to, for example, sarcopenia, and show a positive relationship with cognitive performance [5], [7], [9]. For the cognitive domain, we included tests assessing global cognitive performance, memory, processing speed and various aspects of executive function. A proper functioning of these cognitive domains is important for our functioning. Moreover, memory and executive function are well-documented in terms of age-related decline [25], [26], and have a reported positive relationship with physical performance [5], [9]. We addressed the following questions in this study: (1) Which physical performance measures are the strongest predictors of cognitive performance? (2) Do different physical performance tests predict different aspects of cognitive performance? (3) Do gender and age moderate the association between physical and cognitive performance?

## Methods

### Ethics statement

The local medical ethical committee of the university medical center of Groningen, the Netherlands, approved the study and all participants provided a signed informed consent prior to the assessments. The study was conducted in accordance with the Declaration of Helsinki (59^th^ Amendment).

### Subjects and Design

**Figure 1:**
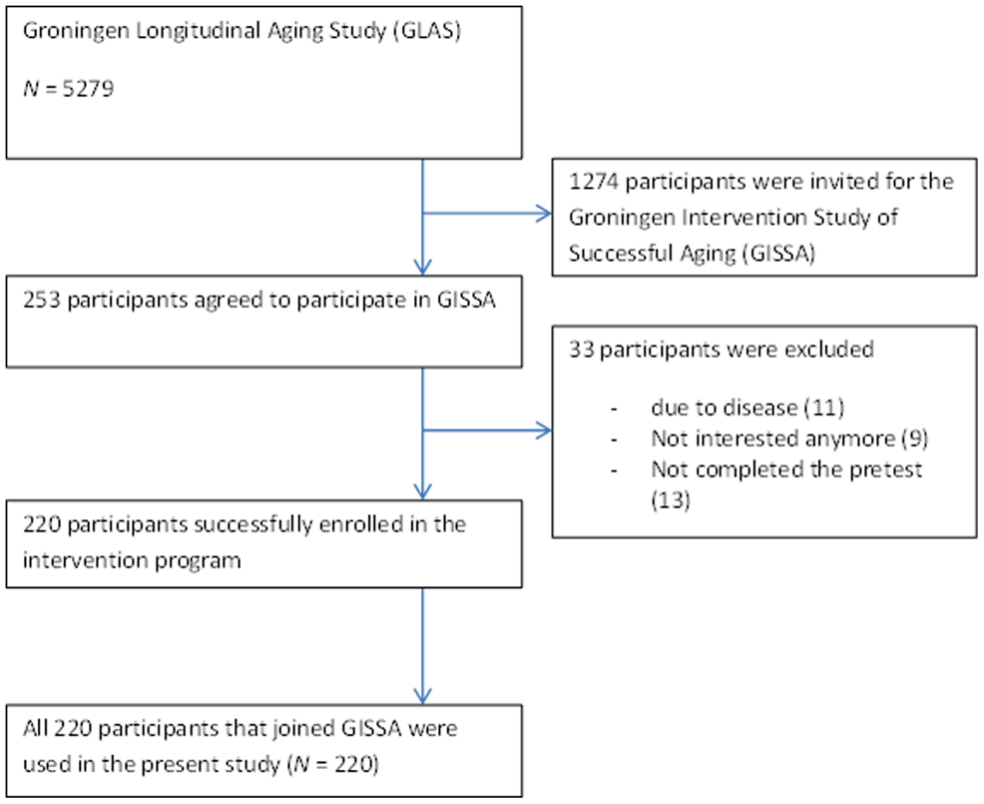
Flowchart specifying the participant selection.

In this study, 220 older community-dwelling adults, with a mean age of 74 years (SD = 5.6; range 65–92) participated. The participants were drawn from the baseline measurement of the Groningen Intervention Study for Successful Aging [27], an intervention study with participants of 65 years and older, which in turn recruited its participants from a longitudinal cohort study [28]. A flow chart illustrating the participant selection procedure is presented in Figure 1. Inclusion criteria for the Groningen Intervention Study for Successful Aging and therefore our study were: (1) being older than 65, (2) having no cognitive decline, as indicated by a score of 24 or lower on the MMSE [6], [29], (3) not exceeding the physical activity guidelines set by the American College of Sports and Medicine for healthy older adults, i.e. five times a week, 30 minutes of moderate intensity physical activity [27], and (4) having no medical condition preventing participation in a physical intervention study (e.g., severe heart problems). All 220 participants included in our study performed the neuropsychological and physical performance tests. The 33 participants who were excluded (see Figure 1) had withdrawn prior to the pretest or had only performed the neuropsychological or physical performance tests. Table 1 shows the characteristics of the participants. Women had a significantly lower level of education and income than men. Both men and women reported low numbers of chronic medical conditions, but women reported significant more chronic medical conditions than men. Women suffered significantly more from high blood pressure, rheumatoid arthritis, and neurologic diseases. Women also reported significantly more use of a walking aid.

**Table 1:** Participant characteristics.

	Men	Women	*p*-value assessing group difference
N	98	122	
Age: Mean (SD)	74.4 (5.57)	73.7 (5.61)	.34
Age: Range	65–89	65–92	
Education: Mean (SD)	4.30 (1.51)	3.94 (1.45)	.08
* Finished primary school or lower*	14.3%	19.7%	
* Lower than finished higher education*	61.2%	66.4%	
* Finished higher education*	24.5%	13.9%	
Income: Mean (SD)	2.72 (.58)	2.31 (.80)	.03
* Below average*	6.1%	17.2%	
* Average*	13.3%	21.3%	
* Above average*	71.4%	41.8%	
Walking Aid: (N)	6	20	.01
Number of medical conditions: Mean (SD)	1.06 (1.03)	1.45 (1.28)	.02
Number of medical conditions: Range	0–5	0–7	

Below average is scored as 1, average is scored as 2, above average is scored as 3. Number of medical conditions is the summarized score of the ICD-10 scores of the participants.

### Potential confounding variables

Various sociodemographic factors which may influence the risk for a decline in cognitive or physical performance (such as education and income) were measured [30]. The level of education was assessed on a seven-point scale suitable for the Dutch education system. The scores range from 1, less than primary school to 7, a university master's degree. Income (after tax) was classified as below average (1), average (2), or above average (3) of the Dutch population according to Statistics Netherlands, an independent government-funded organization [31]. Other possible confounding factors included were the level of anxiety and depression, the amount of time people spent on physical activity in their spare time, and the number of comorbidities. These factors were measured using the scores on the Dutch version of the Hospital Anxiety and Depression Scale for Anxiety and Depression [32], the score on the Minnesota Leisure Time Physical Activity questionnaire (MLTPA) [33], and the number of comorbidities based on the international classification of diseases (these were summed).

### Assessment of cognitive functions

Neuropsychological tests assessed general intelligence and performance on various cognitive domains and the included tests have good reliability and validity [34]–[36]. For all tests, except those using time scores, higher scores indicate a better performance. Each test was administered by trained students from the Center for Human Movement Sciences or the Psychology department.

*Global cognitive performance* was assessed with the Cognitive screening test (CST) [35], an instrument to measure cognitive decline. Scores range from 0 to 20 [35].

*Verbal comprehension* was assessed with the information subtest of the Wechsler adult intelligence scale III (WAIS-III) [37].

*Perceptual organization* was assessed with the matrix reasoning subtest of the WAIS-III [37].

*Memory* was assessed with the Dutch version of the Rey Verbal learning test [38]. This test was used for short-term and long-term memory function. A list of 15 words is presented five times. Short-term memory was assessed from direct recall (score range 0–75), long-term memory from the delayed recall after 15–20 minutes (score range 0–15), followed by a recognition test (score range 0–30).

Several executive functions were assessed: planning, inhibition, and set shifting.

*Planning,* was assessed with the Zoo map, which is a subtest from the Behavioral Assessment of the Dysexecutive Syndrome (BADS) [36]. The Zoo map test required the participant to plan a course through a zoo while adhering to specific rules. Scores range from 0–16.

*Inhibition and processing speed* were assessed with the Stroop test [34], [39]. First, participants had to read a list of one hundred words with the names of four different colors printed in black ink (i.e. Stroop ‘word’). Second, participants had to name the color of one hundred different squares (i.e. Stroop ‘color’) using the same four colors as on card 1. These two cards are thought to mainly measure processing speed. Finally, as a measure of inhibition, one hundred words of the same four colors were presented in different colors of ink and participants had to name the color the words were printed in (i.e. Stroop ‘word-color’). A difference score was also calculated and measures inhibition (Δ Stroop: time on Stroop ‘word-color’ minus time on Stroop ‘color’). The time to complete a card was noted in seconds and lower scores indicate a better performance.

*Visuomotor attention and set-shifting* were assessed with the Trail Making Test (TMT) [34], [40]. In part A (measuring visuomotor attention) of the TMT, participants had to draw a line between encircled numbers. In part B, they had to alternate between circles with numbers and letters (1-A, 2-B) to assess set-shifting. A difference score was calculated to represent a measure of set-shifting (ΔTMT: time on TMT B minus time on TMT A). The time to complete the tasks was noted in seconds and lower scores indicate a better performance.

*Processing speed* was measured with the digit symbol substitution test (DSST) [37]. The DSST is a paper and pencil test of psychomotor performance. The test consists of a key grid of numbers (0–9) with corresponding symbols, followed by the test section. In the test section rows of numbers with empty spaces below them are provided and participants have to fill in as many corresponding symbols as possible in 120 seconds [37]. The score was equal to the number of correctly filled boxes after 120 seconds.

### Assessment of physical performance

The selected tests represent different aspects of physical and motor performance. The validity and reliability of these tests are acceptable and have been reported previously [41]. For all tests higher scores indicate a better performance.

*Grip strength* was assessed with a Jamar® hand dynamometer. Participants grasped the dynamometer with the preferred hand with the arm at the side of the body and the palm toward the thigh. Subjects were instructed to squeeze the dynamometer handle as hard as possible; the highest score (in kg) of three trials was recorded.

*Quadriceps strength* was assessed with a custom built dynamometer, the Quadriso-tester [42]. The favored leg is tested. Participants sat on a chair with knees in 90° flexion. The load cell was located in an ankle cuff that was placed above the ankle joint of the dominant leg and the participants were instructed to press as hard as possible for 3 seconds. The highest score of three trials was recorded.

*Balance* was assessed on an unstable platform that could tilt sideways [41]. For 30 seconds the subjects had to keep the platform in a position so that edge of the platform would not contact the floor. Ground contact was measured with pressure sensors. The time (s) in balance (i.e. the edges of the platform were not in contact with the floor) was recorded. The trial (out of three) with the longest time was selected as a measure of balance performance.

*Functional reach* measures the maximal reach when standing. A subject reaches forward with the dominant arm, having the hand in a fist, the feet maintaining a fixed base of support, while sliding a measurement cube forward over a metal bar. The maximum distance (cm) was recorded and divided by the length of the participant. The trial (out of three) with the longest reach was selected as a measure of functional reach.

*Walking speed* was assessed over a 15 meter long level surface course. Participants were instructed to walk at a self-selected pace. The average duration in seconds of two trials was used as a measure of walking speed.

*Walking endurance* was assessed on an indoor walking track. Participants had to perform as many laps as possible on a 50-m-long rectangle track. Walking speed was increased by 1 km/h every 3 min, starting at a speed of 4 km/h and ending at a speed of 7 km/h. There were beeps between the four corners of the rectangle to guide the requested pace. Participants should reach the next corner on the following beep, if they failed to reach the corner in time the test was finished. The number of completed trajectories (i.e. a side of the rectangle) was recorded [43].

### Statistical analyses

SPSS 18.01 and R 2.10.1 were used to analyze the data. Means and standard deviations were calculated for neuropsychological and physical performance scores. A Student's *t*-test and the chi-square test were used to determine differences between men and women. Grip strength, leg strength, gait speed, the trail making test, and the Stroop test were positively skewed and were, therefore, log-transformed.

The scores of all numeric variables were standardized by converting them to *z*-scores in order to facilitate comparison. The physical and cognitive test scores were inverted when a lower score indicated a higher performance (i.e. in this way a higher score always corresponds to a higher performance). Whenever physical measures correlated close to *r = *0.7, we combined them in a single measure (i.e. by averaging their z-scores) to prevent multicollinearity.

Consequently, we combined walking speed and endurance (*r = *.67, *p = *.000, *N = *220) into a variable Walking ability, and grip strength and quadriceps strength (*r = *.75, *p = *.000, *N = *220) into a variable Strength. In sum, four physical factors were identified: Strength, Walking ability, Balance, and Functional reach. As the scores on grip strength, leg strength, walking speed and endurance were highly gender-dependent (with men having higher scores than women), we corrected those scores for men downwards before calculating the z-scores (i.e. new score men  =  (mean score women/mean score men) * original score men).

As the cognitive domain contained more tests than the physical domain, and there was substantial overlap between the tests, we conducted an exploratory factor analysis (maximum likelihood estimation with oblique rotation) to identify variables which could be grouped. In this way, the complexity of the dataset was reduced substantially as it yielded fewer measurement points per subject. A factor loading of .32 was set as the minimum to be printed in the output of the factor analysis [44]. Each neuropsychological test was uniquely assigned to the factor where it had the highest loading. Subsequently, for each of the resulting factors (described in the following section) the corresponding cognitive score was calculated as the average of the standardized scores of the neuropsychological tests linked to the factor. Since every participant had scores on multiple different cognitive factors, we used linear mixed-effects regression modeling (LMER) with participant as a random-effect factor [45] to take the structural variation linked to each participant into account (i.e. participants who scored high on one cognitive factor are more likely to score high on another cognitive factor). In the analysis, the cognitive score was used as the dependent variable. By including the type of cognitive factor in our model, we were able to assess the precise effect of the physical performance measurements for each individual cognitive factor (i.e. we assessed the possible interaction between cognitive factor and each of the physical performance predictors).

The significance of fixed-effect predictors was evaluated by means of the *t*-test for the coefficients, in addition to model comparison likelihood ratio tests and AIC (Akaike Information Criterion; [46]). When the dataset is large enough (as in our case) the *t-*distribution resembles the normal distribution and variables are significant when their absolute *t-*value is at least 1.65 (when a directional hypothesis is used, i.e. applying a one-tailed test) or at least 2 (for a two-tailed test). As there is a large amount of evidence supporting a positive association between physical performance and cognitive performance we only used a one-tailed test for assessing the significance of the physical measures.

In addition, we conducted model comparison tests to assess if each predictor or interaction significantly improved the model by comparing the log-likelihood and AIC (Akaike Information Criterion) values of the more complex model (i.e. including the additional predictor) to a baseline model (the same as the more complex model, but excluding the additional predictor). A lower AIC (or higher log-likelihood) indicates a better model [46]. On the basis of the AIC values the evidence ratio can be calculated which expresses the relative probability that the model with the lowest AIC is more likely to provide a more precise model of the data. The evidence ratio is exponentially related to the AIC difference. For example if the AIC difference is 2 (generally used as the minimum required reduction), then the model is 2.7 times more likely to provide a precise model of the data, whereas an AIC difference of 8 indicates that the model with the lowest AIC is 54.6 times more likely to provide a precise model of the data [46].

### Missing values

Inspection of our dataset revealed only a few missing values. Less than 1% percent of the data was missing with respect to the physical performance test data, whereas only 0.1% of the data was missing for all neuropsychological tests. The limited amount of missing data is ‘trivial’ [47] and we used regression substitution to replace these missing values. This method is preferred over replacing missing values by their mean or deleting the cases with missing values [48]. Whereas multiple imputation [49] is the preferred method to deal with missing values, the amount of missing data was very limited in this study and we therefore opted for the simpler method of regression substitution.

## Results

Table 2 presents the descriptive data for the neuropsychological and physical performance measures. Women performed significantly better than men with respect to Stroop ‘color’, Stroop ‘word-color’, and verbal memory. Men performed significantly better with respect to the WAIS subtest information, and also across the complete physical domain: men were stronger, faster, and had greater endurance than women. Only functional reach, after correcting for body height, was not significantly different between men and women.

**Table 2:** Means and standard deviations (SD) for the neuropsychological and physical performance tests.

	Men	Women	*p*-value assessing difference between men and women	All participants
***Neuropsychological tests***				
CST	19.17 (1.52)	19.05 (1.15)	.51	19.11 (1.33)
WAIS information	16.17 (5.32)	13.23 (4.89)	<.001	14.54 (5.28)
WAIS matrix reasoning	12.23 (5.47)	12.28 (5.36)	.95	12.26 (5.40)
15 WT direct recall	31.47 (9.51)	38.63 (9.60)	<.001	35.44 (10.18)
15 WT delayed recall	6.49 (2.70)	8.07 (2.55)	<.001	7.37 (2.73)
15 WT recognition	27.15 (3.47)	28.53 (2.03)	<.001	27.92 (2.84)
Zoo map 1	.06 (4.75)	−0.97 (4.83)	.12	−.51 (4.81)
TMT B (s)^a^	126.76 (59.13)	127.36 (62.67)	.94	127.09 (60.98)
Δ TMT (s)^a^	73.52 (44.93)	74.77 (53.89)	.85	74.21 (49.99)
Stroop ‘word-color’ (s)^a^	137.39 (49.72)	122.98 (39.31)	.02	129.40 (44.72)
Δ Stroop (s)^a^	66.74 (39.37)	58.21 (34.00)	.09	62.01 (36.65)
TMT A (s)^a^	53.23 (21.61)	52.59 (18.77)	.81	52.88 (20.04)
DSST (score)	45.43 (14.09)	45.70 (11.58)	.87	45.58 (12.73)
Stroop ‘word’ (s)^a^	53.31 (11.10)	52.25 (9.90)	.46	52.72 (10.44)
Stroop ‘color’ (s)^a^	71.37 (16.97)	64.77 (13.21)	.001	67.71 (15.32)
***Physical performance tests***				
Grip strength (kg)	39.56 (9.21)	23.59 (5.48)	<.001	30.70 (10.84)
Quadriceps strength (kg)	41.17 (14.90)	21.19 (7.32)	<.001	30.09 (15.07)
Balance (*.3)	68.98 (9.73)	67.84 (9.76)	.39	68.35 (9.74)
Functional reach (cm/length in cm)	.21(0.04)	.20 (0.04)	.73	.20 (0.04)
Walking speed (s)^a^	20.92 (4.57)	23.37 (7.79)	<.001	22.28 (6.65)
Endurance	34.73 (16.79)	24.02 (13.06)	<.001	28.79 (15.74)

a a lower score indicates better performance; CST, cognitive screening test; WAIS, Wechsler Adult Intelligence Scale; 15 WT, Fifteen word test; TMT, trail making test; ΔTMT, TMT B – TMT A; Δ Stroop, Stroop ‘word-color’ – Stroop ‘color’; DSST, digit symbol substitution test.

The factor analysis of all 15 cognitive tests revealed seven factors for the cognitive performance tests. The seven factors were: verbal attention (Stroop ‘word’), visual attention (Stroop ‘color’), visuomotor attention (TMT-A), set-shifting (TMT B and Δ TMT), inhibition (Stroop ‘word-color’ and Δ Stroop), memory (direct recall, delayed recall, and recognition), and intelligence (WAIS subtest information, WAIS subtest matrices, and CST). As zoo time and the digit symbol substitution test did not reach the loading threshold of .32 for any of the factors, we excluded both from the analysis. Table 3 shows the loadings.

**Table 3:** Results of the factor analysis (using oblique rotation).

	Factor 1	Factor 2	Factor 3	Factor 4	Factor 5	Factor 6	Factor 7
TMT-B	0.74				0.36		
TMT Delta	1.13						
15WT Dir recall		0.79					
15WT Del recall		0.90					
15 WT Rec		0.58					
Stroop ‘word-color’			0.71			0.51	
Stroop Delta			1.09				
WAIS information				1.05			
WAIS matrices				0.37			
CST				0.39			
TMT A					1.11		
Stroop ‘word’							0.89
Stroop ‘color’						0.87	
DSST							
BADS Zoo test							
SS loadings	1.88	1.78	1.75	1.53	1.49	1.25	0.95
Proportion Var	0.13	0.12	0.12	0.10	0.10	0.08	0.06
Cumulative Var	0.13	0.24	0.36	0.46	0.56	0.65	0.71

Test of the hypothesis that 7 factors are sufficient. The chi square statistic is 17.71 on 21 degrees of freedom. The *p*-value is 0.6.

TMT, Trail Making Test; TMT Delta, TMT B – TMT A; 15WT, fifteen word test; Dir, direct; Del, delayed; Rec, recognition; Stroop Delta, Stroop ‘word-color’ – Stroop ‘color’; WAIS, Wechsler Adult Intelligence Scale; CST, Cognitive Screenings Test; DSST, Digit Symbol Substitution, Test; BADS, Behavioural Assessment of Dysexecutive Syndrome; Var, Variance.

### Prediction of cognitive performance by physical performance

Table 4 shows the best mixed-effects regression model (explained variance: 38.2%). This model shows that age (*β = *−.15, *t = *−3.98) and being male (*β = *−.23, *t = *−3.44) have a negative impact on all cognitive factors, whereas education (*β = *.21, *t = *6.33) has a positive effect on these factors. The other potentially confounding variables (i.e. income, comorbidity, depression, anxiety, walking aid and the score on the MLTPA) did not reach significance by themselves or in interaction with any other variables and were therefore not included in the model.

**Table 4:** Linear mixed effects regression model predicting cognitive performance.

Fixed effects	Estimate	Std. Error	*t*-value	*p-value*
(Intercept)	0.09946	0.04431	2.245	<.05
Age	−0.15174	0.03813	−3.980	<.01
Education	0.21125	0.03336	6.333	<.01
Male	−0.23001	0.06679	−3.444	<.01
Strength^a^	0.07180	0.03897	1.842	<.05
Balance*Female	0.06152	0.04661	1.320	.09
Balance*Male	0.16405	0.05172	3.172	<.01
Walking ability^a^ * Female	0.09238	0.04840	1.909	<.05
Walking ability^a^ * Male	0.24888	0.05933	4.195	<.01

a Scores of men were normalized to be comparable with women's scores: see text for details.

Physical predictors of cognitive performance significantly improving the fit of the model were walking ability (*β = .*15, *t = *3.57; Table 4 shows the effect moderated by gender), balance (*β = .*11, *t = *3.00; Table 4 shows the effect moderated by gender) and strength (*β = .*07, *t = *1.84). Functional reach did not reach significance and was excluded from the final model.

The results show that there was no variation in the effect of the physical performance measures on the different factors of cognitive performance. The model did not improve by allowing for a varying effect of the physical performance predictors on each individual cognitive factor (i.e. no interaction reduced the AIC compared to the simpler model with at least 2).

Finally, the model shows a specific interaction between balance and gender and walking ability and gender. For balance, there appears to be no significant effect for women (*β = *.06, *t = *1.32), but a clear significant effect for men (*β = *.16, *t = *3.17). For walking ability, only a small significant effect could be observed for women (*β = *.09, *t = *1.91), but a more pronounced effect for men (*β = *.24, *t = *4.20). No other significant interactions with age or gender were found. Note that the inclusion of the variable indicating that the participant is male (1, or not: 0) improves the model and does not alter the moderating effect of gender on balance and walking ability.

To illustrate the contribution of each predictor (or interaction) to the fit of the model, Table 5 shows the increase in goodness of fit when adding each predictor to the simpler model without the predictor. Given that each variable improves the fit significantly, as can be seen by the log-likelihood ratio test and the decrease in AIC (and associated high evidence ratios), the inclusion of each of the variables reported in this section is warranted.

**Table 5:** Goodness of fit of the fixed-effect factors of the model.

Additional fixed effects	Log –likelihood increase	AIC decrease	Evidence ratio	Likelihood ratio test	Additional degrees of freedom
Random intercept only					
+ Education	10.7	9.5	115.6	*P*<.0001	1
+ Age	25.9	49.8	>1000	*P*<.0001	1
+ Male	0.2	5.6	16.4	*p = *.0059	1
+ Strength	7.2	12.3	478.7	*p = *.0002	1
+ Balance*Male	7.3	10.5	190.6	*p = *.0007	2
+ Walking ability *Male	9.5	15.1	>1000	*P*<.0001	2

Each row specifies the significant increase in goodness of fit obtained by adding the current predictor to the model including all preceding predictors. AIC: Akaike Information Criterion.

## Discussion

The goal of the present study was threefold. First, to determine which domains of physical performance are the strongest predictors of cognitive performance. **S**econd, to identify whether these physical predictors vary for different aspects of cognitive performance, and third, to determine whether age and gender moderate the relation between physical and cognitive performance.

### The strongest physical predictors of cognitive performance

In our study muscle strength and a gender-moderated effect of balance and walking ability were significant predictors of cognitive performance. The predictive value of walking ability, balance and muscle strength for different cognitive tests, such as the TMT (set-shifting), MMSE (global cognition), and Stroop (inhibition) has been observed previously [5], [50]–[54]. Moreover, in the recently published ‘central benefit model’ of Liu-Ambrose and colleagues [55] the importance of the association between walking ability (gait speed), balance and executive functions is postulated as well. For example, falls are not only related to a decline in gait, balance, and muscle strength, but also to a decline in executive functions [55]. Gait is not a simple motor task for older adults. With aging, gait increasingly demands cognitive control [56]. Gait speed, an important component of gait, is associated with executive functions (Stroop test) and also with other cognitive performance such as global cognitive functioning (MMSE) [52], [53], [57]. It has been argued that a higher gait speed increases the cerebral blood flow especially in the prefrontal cortex, a brain region that plays a crucial role in executive functions [58], [59].

Moreover, to be able to maintain balance it is important to have the capability to activate muscles properly, to respond to balance threats, and to possess sufficient levels of muscle strength [60]. Such a role of strength and balance in physical performance might explain why both are predictive of performances across different cognitive components. More specifically, the balance task used in this study also appeals to the executive functions, such as inhibition (i.e. not being distracted by noise) and cognitive flexibility (i.e. being able to compensate for errors).

As strength, walking ability, and balance can be trained in older adults [61]–[63], future studies that focus on the causal relationship between these physical domains and cognitive performance are necessary.

Besides the physical performance measures, several other variables were significant predictors of cognitive performance. Not surprisingly, older participants and participants with a lower education level showed reduced cognitive performance compared to younger and higher educated participants. In addition, men showed lower cognitive performance than women, which is in line with previous findings [18], [19].

### Physical performance and different aspects of cognitive performance

In our study we did not find a differential effect of the physical performance measures balance, strength, and walking ability on the different domains of cognitive performance. This finding might suggest that the link between physical and cognitive performance is relatively similar across cognitive domains for healthy older adults. However, as we did not assess all cognitive domains (e.g., non-verbal memory and planning), further studies are needed to assess if these results also extend to the other cognitive domains.

### The moderating effect of gender and age on the association between physical and cognitive performance

In line with other studies that detected gender differences in cognitive decline and physical decline [11], [14], [16]–[19], we identified a moderating effect of gender on the relationship between cognitive performance and the physical measures of walking ability and balance. For men, both physical measures strongly predicted cognitive performance, but for women only walking ability was a significant predictor of cognitive performance (albeit more reduced than for men).

It is possible that differences in brain morphology between men and women [11]–[14] contribute to these gender effects, or that these sex differences are caused by different metabolic and hormonal responses between men and women [64]. We therefore recommend that future studies specifically test for a possible gender effects.

There is strong evidence for a temporal dissociation between the decline in cognition, muscle strength, and balance [4], [20], [21]. We expected that age would influence the association between physical and cognitive performance, but we did not find such a moderating effect. The lack of additional interaction effects was not in line with our expectations and previously reported age effects [65]. Perhaps participation bias might have attenuated the expected age interaction effects, as very fit participants were excluded by design and many older adults with cognitive or physical difficulties normally refrain from participating in such studies [43].

### Frailty

Our study consisted of relatively healthy elderly. Given that the number of elderly increases rapidly in the Netherlands [66], the number of frail elderly will probably increase even more in the following decades [67]. Especially frail elderly are at risk for adverse events such as falls, hospital admission and cognitive decline [68]. Although our present findings (i.e. the link between physical and cognitive performance measures) cannot be generalized to frail elderly (see below), they do fit the discussion about the concept of frailty. Our findings support the idea of Rockwood and colleagues [69] that frailty is an accumulation of deficits, and should not only consist of physical parameters [70] but also of other parameters such as cognitive performance.

### Limitations

The present study also has several limitations. First, this is a cross-sectional study and therefore conclusions about causality cannot be drawn. Second, the participants in this study (aged over 65) formed a rather homogeneous group. The healthiest elderly were not included and their non-responding peers, we suspect, would have been more frail (i.e. having lower physical and cognitive performance) than those who were included. These biases obviously restrict the generalizability of our results to other subgroups. Although the physical and neuropsychological test scores were similar to those reported in other cross-sectional studies in healthy older adults [9], [53], [57], [71]–[74], and similar prediction accuracies were found compared with previous studies [9], [75], further studies need to assess if our results (presented in Table 4) are valid for other subgroups, such as participants suffering from cognitive decline, frail participants, or participants under the age of 65.

## Conclusions

To the best of our knowledge, this is the first comprehensive assessment of the relationship between physical and cognitive performance in healthy older adults. We identified walking ability, balance and strength to be significant predictors of cognitive performance. Our finding that walking ability and balance are stronger predictors of cognitive performance for men than women, suggests that the effect of strength and balance training in older men might have a larger impact on cognitive performance than for women.

Future studies, however, need to investigate a possible causal relationship between physical and cognitive performance and also focus on the generalizability of these results to other groups, such as frail older people and patients with dementia.

## References

[1] Mura T, Dartigues JF, Berr C (2010) How many dementia cases in france and europe? alternative projections and scenarios 2010–2050. Eur J Neurol 17: 252–259. 19796284 10.1111/j.1468-1331.2009.02783.x PMC2925047

[2] Kuo HK, Leveille SG, Yu YH, Milberg WP (2007) Cognitive function, habitual gait speed, and late-life disability in the national health and nutrition examination survey (NHANES) 1999–2002. Gerontology 53: 102–110. 17090975 10.1159/000096792 PMC2365496

[3] Lynch GS (2004) Tackling australia's future health problems: Developing strategies to combat sarcopenia – age-related muscle wasting and weakness. Intern Med J 34: 294–296. 15151679 10.1111/j.1444-0903.2004.00568.x

[4] Salthouse TA (2009) When does age-related cognitive decline begin? Neurobiol Aging 30: 507–514. 19231028 10.1016/j.neurobiolaging.2008.09.023 PMC2683339

[5] Fitzpatrick AL, Buchanan CK, Nahin RL, Dekosky ST, Atkinson HH, et al (2007) Associations of gait speed and other measures of physical function with cognition in a healthy cohort of elderly persons. J Gerontol A Biol Sci Med Sci 62: 1244–1251. 18000144 10.1093/gerona/62.11.1244

[6] Folstein MF, Folstein SE, McHugh PR (1975) “Mini-mental state”. A practical method for grading the cognitive state of patients for the clinician. J Psychiatr Res 12: 189–98. 1202204 10.1016/0022-3956(75)90026-6

[7] Wang L, Larson EB, Bowen JD, van Belle G (2006) Performance-based physical function and future dementia in older people. Arch Intern Med 166: 1115–1120. 16717174 10.1001/archinte.166.10.1115

[8] Rosano C, Sigurdsson S, Siggeirsdottir K, Phillips CL, Garcia M, et al (2010) Magnetization transfer imaging, white matter hyperintensities, brain atrophy and slower gait in older men and women. Neurobiol Aging 31: 1197–1204. 18774624 10.1016/j.neurobiolaging.2008.08.004 PMC2873052

[9] Huh Y, Yang EJ, Lee SA, Lim JY, Kim KW, et al (2011) Association between executive function and physical performance in older korean adults: Findings from the korean longitudinal study on health and aging (KLoSHA). Arch Gerontol Geriatr 52: e156–61. 21075462 10.1016/j.archger.2010.10.018

[10] Miller DI, Taler V, Davidson PS, Messier C (2012) Measuring the impact of exercise on cognitive aging: Methodological issues. Neurobiol Aging 33: 622.e29–622.e43. 10.1016/j.neurobiolaging.2011.02.020 21514694

[11] Rubia K, Hyde Z, Halari R, Giampietro V, Smith A (2010) Effects of age and sex on developmental neural networks of visual-spatial attention allocation. Neuroimage 51: 817–827. 20188841 10.1016/j.neuroimage.2010.02.058

[12] Gur RC, Mozley PD, Resnick SM, Gottlieb GL, Kohn M, et al (1991) Gender differences in age effect on brain atrophy measured by magnetic resonance imaging. Proc Natl Acad Sci U S A 88: 2845–2849. 2011592 10.1073/pnas.88.7.2845 PMC51336

[13] Raz N, Gunning FM, Head D, Dupuis JH, McQuain J, et al (1997) Selective aging of the human cerebral cortex observed in vivo: Differential vulnerability of the prefrontal gray matter. Cereb Cortex 7: 268–282. 9143446 10.1093/cercor/7.3.268

[14] Misteli M, Duschek S, Richter A, Grimm S, Rezk M, et al (2011) Gender characteristics of cerebral hemodynamics during complex cognitive functioning. Brain Cogn 76: 123–130. 21420774 10.1016/j.bandc.2011.02.009

[15] Narici MV, Maffulli N (2010) Sarcopenia: Characteristics, mechanisms and functional significance. Br Med Bull 95: 139–159. 20200012 10.1093/bmb/ldq008

[16] Desrosiers J, Hebert R, Bravo G, Rochette A (1999) Age-related changes in upper extremity performance of elderly people: A longitudinal study. Exp Gerontol 34: 393–405. 10433393 10.1016/s0531-5565(99)00018-2

[17] Wu Y, Zhang D, Pang Z, Oksuzyan A, Jiang W, et al. (2011) Gender-specific patterns in age-related decline in general health among danish and chinese: A cross-national comparative study. Geriatr Gerontol Int. 10.1111/j.1447-0594.2011.00784.x 22212497

[18] Maylor EA, Reimers S, Choi J, Collaer ML, Peters M, et al (2007) Gender and sexual orientation differences in cognition across adulthood: Age is kinder to women than to men regardless of sexual orientation. Arch Sex Behav 36: 235–249. 17351741 10.1007/s10508-006-9155-y

[19] Zelinski EM, Stewart ST (1998) Individual differences in 16-year memory changes. Psychol Aging 13: 622–630. 9883462 10.1037//0882-7974.13.4.622

[20] Era P, Heikkinen E, Gause-Nilsson I, Schroll M (2002) Postural balance in elderly people: Changes over a five-year follow-up and its predictive value for survival. Aging Clin Exp Res 14: 37–46. 12475132

[21] Waters DL, Baumgartner RN, Garry PJ (2000) Sarcopenia: Current perspectives. J Nutr Health Aging 4: 133–139. 10936899

[22] Milanovic Z, Pantelic S, Trajkovic N, Sporis G, Kostic R, et al (2013) Age-related decrease in physical activity and functional fitness among elderly men and women. Clin Interv Aging 8: 549–556. 23723694 10.2147/CIA.S44112 PMC3665513

[23] Tinetti ME, Baker DI, McAvay G, Claus EB, Garrett P, et al (1994) A multifactorial intervention to reduce the risk of falling among elderly people living in the community. N Engl J Med 331: 821–827. 8078528 10.1056/NEJM199409293311301

[24] Samson MM, Crowe A, de Vreede PL, Dessens JA, Duursma SA, et al (2001) Differences in gait parameters at a preferred walking speed in healthy subjects due to age, height and body weight. Aging (Milano) 13: 16–21. 11292147 10.1007/BF03351489

[25] Weinstein AM, Voss MW, Prakash RS, Chaddock L, Szabo A, et al (2012) The association between aerobic fitness and executive function is mediated by prefrontal cortex volume. Brain Behav Immun 26: 811–819. 22172477 10.1016/j.bbi.2011.11.008 PMC3321393

[26] Raz N, Lindenberger U, Rodrigue KM, Kennedy KM, Head D, et al (2005) Regional brain changes in aging healthy adults: General trends, individual differences and modifiers. Cereb Cortex 15: 1676–1689. 15703252 10.1093/cercor/bhi044

[27] van Heuvelen MJ, Hochstenbach JB, Brouwer WH, de Greef MH, Zijlstra GA, et al (2005) Differences between participants and non-participants in an RCT on physical activity and psychological interventions for older persons. Aging Clin Exp Res 17: 236–245. 16110738 10.1007/BF03324603

[28] Kempen GI, Jelicic M, Ormel J (1997) Personality, chronic medical morbidity, and health-related quality of life among older persons. Health Psychol 16: 539–546. 9386999 10.1037//0278-6133.16.6.539

[29] Boban M, Malojcic B, Mimica N, Vukovic S, Zrilic I, et al (2012) The reliability and validity of the mini-mental state examination in the elderly croatian population. Dement Geriatr Cogn Disord 33: 385–392. 22814030 10.1159/000339596

[30] Black SA, Rush RD (2002) Cognitive and functional decline in adults aged 75 and older. J Am Geriatr Soc 50: 1978–1986. 12473009 10.1046/j.1532-5415.2002.50609.x

[31] Statistics Netherlands (2012) Verschil lonen en uitkeringen laatste twintig jaar fors toegenomen. 2012.

[32] Spinhoven P, Ormel J, Sloekers PP, Kempen GI, Speckens AE, et al (1997) A validation study of the hospital anxiety and depression scale (HADS) in different groups of dutch subjects. Psychol Med 27: 363–370. 9089829 10.1017/s0033291796004382

[33] Folsom AR, Jacobs DR Jr, Caspersen CJ, Gomez-Marin O, Knudsen J (1986) Test-retest reliability of the minnesota leisure time physical activity questionnaire. J Chronic Dis 39: 505–511. 3722314 10.1016/0021-9681(86)90195-5

[34] Lezak MD, Howieson DB, Loring DW (2004) Neuropsychological assessment (4th ed). New York, USA: Oxford University Press.

[35] Graaf A, Deelman BG. (1991) Cognitieve screening test. Lisse, The Netherlands: Swets & Zeitlinger.

[36] Wilson BA, Alderman N, Burgess PW, Emslie H, Evans JJ (1997) Behavioural assessment of the dysexecutive syndrome. Bury st. Edmuns, England: Thames Valley Test Company.

[37] Wechsler D (2004) Wechsler memory scale. Madrid, Spain: TEA.

[38] Schmidt M (1996) Rey auditory and verbal learning test: A handbook. Los Angeles, USA: Western psychological services.

[39] Goldon CJ (1978) Stroop color and word test: A manual for clinical and experimental use. In: Anonymous Chicago, USA: Stoelting.

[40] Reitan RM, Wolfson D (1985) The halstead-reitan neuropsycholgical test battery. Tucson, AZ, USA: Neuropsychology Press.

[41] Van Heuvelen MJ, Kempen GI, Ormel J, Rispens P (1998) Physical fitness related to age and physical activity in older persons. Med Sci Sports Exerc 30: 434–441. 9526891 10.1097/00005768-199803000-00015

[42] Verkerke GJ, Lemmink KA, Slagers AJ, Westhoff MH, van Riet GA, et al (2003) Precision, comfort and mechanical performance of the quadriso-tester, a quadriceps force measuring device. Med Biol Eng Comput 41: 283–289. 12803292 10.1007/BF02348432

[43] van Heuvelen MJ, Hochstenbach JB, Brouwer WH, de Greef MH, Scherder E (2006) Psychological and physical activity training for older persons: Who does not attend? Gerontology 52: 366–375. 16905888 10.1159/000094986

[44] Tabachnik BG, Fidell LS (2001) Using multivariate statistics. USA: Needham Heights.

[45] Pinheiro JC, Bates DM.(2000) Mixed-effects modesl in S and S-PLUSS. New York: Springer.

[46] Aikaike H (1974) A new look at the statistical model identification. EEE Transactions on Automatic Control 19: 716.

[47] Acuna E, Rodriguez C (2004) The treatment of missing values and its effect in the classifier accuracy. In: Anonymous Classification, clustering and datamining applications. 639.

[48] Cleophas EP, Cleophas TJ (2011) Clinical research: A novel approach to regression substitution for handling missing data. Am J Ther. 10.1097/MJT.0b013e3181ff7a7b 21866042

[49] Rubin DB. (1987) Multiple imputation for nonresponse in surveys. New York: Wiley.

[50] Ble A, Volpato S, Zuliani G, Guralnik JM, Bandinelli S, et al (2005) Executive function correlates with walking speed in older persons: The InCHIANTI study. J Am Geriatr Soc 53: 410–415. 15743282 10.1111/j.1532-5415.2005.53157.x

[51] Malmstrom TK, Wolinsky FD, Andresen EM, Miller JP, Miller DK (2005) Cognitive ability and physical performance in middle-aged african americans. J Am Geriatr Soc 53: 997–1001. 15935023 10.1111/j.1532-5415.2005.53318.x

[52] McGough EL, Kelly VE, Logsdon RG, McCurry SM, Cochrane BB, et al (2011) Associations between physical performance and executive function in older adults with mild cognitive impairment: Gait speed and the timed “up & go” test. Phys Ther 91: 1198–1207. 21616934 10.2522/ptj.20100372 PMC3145896

[53] Rosano C, Simonsick EM, Harris TB, Kritchevsky SB, Brach J, et al (2005) Association between physical and cognitive function in healthy elderly: The health, aging and body composition study. Neuroepidemiology 24: 8–14. 15459503 10.1159/000081043

[54] Voelcker-Rehage C, Godde B, Staudinger UM (2010) Physical and motor fitness are both related to cognition in old age. Eur J Neurosci 31: 167–176. 20092563 10.1111/j.1460-9568.2009.07014.x

[55] Liu-Ambrose T, Nagamatsu LS, Hsu CL, Bolandzadeh N (2013) Emerging concept: ‘central benefit model’ of exercise in falls prevention. Br J Sports Med 47: 115–117. 22522589 10.1136/bjsports-2011-090725 PMC5226845

[56] Hausdorff JM, Yogev G, Springer S, Simon ES, Giladi N (2005) Walking is more like catching than tapping: Gait in the elderly as a complex cognitive task. Exp Brain Res 164: 541–8. 15864565 10.1007/s00221-005-2280-3

[57] Netz Y, Dwolatzky T, Zinker Y, Argov E, Agmon R (2011) Aerobic fitness and multidomain cognitive function in advanced age. Int Psychogeriatr 23: 114–124. 20566000 10.1017/S1041610210000797

[58] Sorond FA, Kiely DK, Galica A, Moscufo N, Serrador JM, et al (2011) Neurovascular coupling is impaired in slow walkers: The MOBILIZE boston study. Ann Neurol 70: 213–220. 21674588 10.1002/ana.22433 PMC3152682

[59] Nakamura T, Meguro K, Yamazaki H, Okuzumi H, Tanaka A, et al (1997) Postural and gait disturbance correlated with decreased frontal cerebral blood flow in alzheimer disease. Alzheimer Dis Assoc Disord 11: 132–139. 9305498 10.1097/00002093-199709000-00005

[60] Hawkes TD, Siu KC, Silsupadol P, Woollacott MH (2012) Why does older adults' balance become less stable when walking and performing a secondary task? examination of attentional switching abilities. Gait Posture 35: 159–163. 21964051 10.1016/j.gaitpost.2011.09.001 PMC3251721

[61] Fiatarone MA, O'Neill EF, Ryan ND, Clements KM, Solares GR, et al (1994) Exercise training and nutritional supplementation for physical frailty in very elderly people. N Engl J Med 330: 1769–75. 8190152 10.1056/NEJM199406233302501

[62] Volkers KM, de Kieviet JF, Wittingen HP, Scherder EJ (2012) Lower limb muscle strength (LLMS): Why sedentary life should never start? A review. Arch Gerontol Geriatr 54: 399–414. 21601928 10.1016/j.archger.2011.04.018

[63] Shimada H, Tiedemann A, Lord SR, Suzukawa M, Makizako H, et al (2011) Physical factors underlying the association between lower walking performance and falls in older people: A structural equation model. Arch Gerontol Geriatr 53: 131–134. 21145119 10.1016/j.archger.2010.11.003

[64] Baker LD, Frank LL, Foster-Schubert K, Green PS, Wilkinson CW, et al (2010) Effects of aerobic exercise on mild cognitive impairment: A controlled trial. Arch Neurol 67: 71–79. 20065132 10.1001/archneurol.2009.307 PMC3056436

[65] Colcombe S, Kramer AF (2003) Fitness effects on the cognitive function of older adults: A meta-analytic study. Psychol Sci 14: 125–30. 12661673 10.1111/1467-9280.t01-1-01430

[66] van Duin C, Garssen J (2011) Bevolkingsprognose 2010–2060: Sterkere vergrijzing, langere levensduur.

[67] Slaets JP (2006) Vulnerability in the elderly: Frailty. Med Clin North Am 90: 593–601. 16843764 10.1016/j.mcna.2006.05.008

[68] Shim EY, Ma SH, Hong SH, Lee YS, Paik WY, et al (2011) Correlation between frailty level and adverse health-related outcomes of community-dwelling elderly, one year retrospective study. Korean J Fam Med 32: 249–256. 22745861 10.4082/kjfm.2011.32.4.249 PMC3383131

[69] Rockwood K, Mitnitski A (2007) Frailty in relation to the accumulation of deficits. J Gerontol A Biol Sci Med Sci 62: 722–727. 17634318 10.1093/gerona/62.7.722

[70] Fried LP, Tangen CM, Walston J, Newman AB, Hirsch C, et al (2001) Frailty in older adults: Evidence for a phenotype. J Gerontol A Biol Sci Med Sci 56: M146–56. 11253156 10.1093/gerona/56.3.m146

[71] Rudisill ME, Toole T (1993) Gender differences in motor performance of 50- to 79-year-old adults. Percept Mot Skills 77: 939–947. 8284181 10.2466/pms.1993.77.3.939

[72] Liu-Ambrose T, Davis JC, Nagamatsu LS, Hsu CL, Katarynych LA, et al (2010) Changes in executive functions and self-efficacy are independently associated with improved usual gait speed in older women. BMC Geriatr 10: 25. 20482830 10.1186/1471-2318-10-25 PMC2887871

[73] Anstey KJ, Lord SR, Williams P (1997) Strength in the lower limbs, visual contrast sensitivity, and simple reaction time predict cognition in older women. Psychol Aging 12: 137–44. 9100274 10.1037//0882-7974.12.1.137

[74] Kaushanskaya M, Marian V, Yoo J (2011) Gender differences in adult word learning. Acta Psychol (Amst) 137: 24–35. 21392726 10.1016/j.actpsy.2011.02.002 PMC3080468

[75] Scherder EJ, Eggermont LH, Geuze RH, Vis J, Verkerke GJ (2010) Quadriceps strength and executive functions in older women. Am J Phys Med Rehabil 89: 458–463. 20216058 10.1097/PHM.0b013e3181d3e9f6

